# Characterization of Infiltrating Immune Cells and Secretory or Membrane-Associated Proteins in KRAS Lung Adenocarcinoma

**DOI:** 10.1155/2023/4987832

**Published:** 2023-02-06

**Authors:** Yunyi Bian, Guoshu Bi, Guangyao Shan, Jiaqi Liang, Qihai Sui, Zhengyang Hu, Qun Wang, Yi Zhang, Hong Fan

**Affiliations:** Department of Thoracic Surgery, Zhongshan Hospital, Fudan University, Shanghai, China

## Abstract

**Background:**

This study identified the expression and prognosis significance of secretory or membrane-associated proteins in KRAS lung adenocarcinoma (LUAD) and depicted the characteristics between the immune cell infiltration and the expression of these genes.

**Methods:**

Gene expression data of LUAD samples (*n* = 563) were accessed from The Cancer Genome Atlas (TCGA). The expression of secretory or membrane-associated proteins was compared among the KRAS-mutant, wild-type, and normal groups, as well as the subgroup of the KRAS-mutant group. We identified the survival-related differentially expressed secretory or membrane-associated proteins and conducted the functional enrichment analysis. Then, the characterization and association between their expression and the 24 immune cell subsets were investigated. We also constructed a scoring model to predict KRAS mutation by LASSO and logistic regression analysis.

**Results:**

Secretory or membrane-associated genes with differential expression (*n* = 74) across three groups (137 KRAS LUAD, 368 wild-type LUAD, and 58 normal groups) were identified, and the results of GO and KEGG indicated that they were strongly associated with immune cell infiltrations. Among them, ten genes were significantly related to the survival of patients with KRAS LUAD. The expression of IL37, KIF2, INSR, and AQP3 had the most significant correlations with immune cell infiltration. In addition, eight DEGs from the KRAS subgroups were highly correlated with immune infiltrations, especially TNFSF13B. Using LASSO-logistic regression, a KRAS mutation prediction model based on the 74 differentially expressed secretory or membrane-associated genes was built, and the accuracy was 0.79.

**Conclusion:**

The research investigated the relationship between the expression of KRAS-related secretory or membrane-associated proteins in LUAD patients with prognostic prediction and immune infiltration characterization. Our study demonstrated that secretory or membrane-associated genes were closely associated with the survival of KRAS LUAD patients and were strongly correlated to immune cell infiltration.

## 1. Introduction

Lung cancer has a high mortality and morbidity rate, and its average five-year survival rate is under 15% [1, 2]. Non-small-cell lung cancer (NSCLC) is the most prevalent histological type [3–6], accounting for 85 percent of all lung cancers, and more than 40% of patients are diagnosed with locally progressed or advanced stages, so losing their opportunity to accept surgery. Several clinical studies have proved that immunotherapy, such as anti-programmed cell death protein 1 (PD-1)/programmed cell death-ligand 1 (PD-L1) medicines, has proven efficacious in treating patients with advanced NSCLC [3].

Secreted or membrane-located proteins (SMP) were the critical mediators between tumor and immune-infiltrating cells, which were important for immunotherapy and deserved further study [7]. Programmed cell death protein 1 (PD-1) is one of the secretory or membrane-associated proteins that may be produced in a variety of human cells, including T cells. The tumor microenvironment (TME) is a crucial component in carcinogenesis, and immune evasion is a critical phase in tumor growth, progression, and therapeutic resistance [8]. Infiltrating immune cells are necessary immunotherapy efficiency markers as an essential component of the immune microenvironment [7]. Studying the characterization between secretory or membrane-associated proteins and infiltrating immune cells in the TME is crucial for selecting immunotherapeutic targets [9]. Using the recently published computational approach gene set expansion analysis (GSEA) [9], researchers could evaluate the extent of immune cell infiltration in the immunological microenvironment.

In addition, it remains uncertain whether NSCLC patients with driver mutations will benefit from anti-PD-1/PD-L1 medicines. Kirsten rat sarcoma virus oncogene (KRAS) mutation, occurring in 30% of NSCLC [10], serves a vital role in the development of tumors [9]. Point mutation is the most common way of KRAS mutation, and the most common types are KRAS-G12D mutation (41%), KRAS-G12V mutation (28%), and KRAS-G12C mutation (14%) [11]. In NSCLC, G12C was the most prevalent type [12], which is strongly connected with a poor prognosis [13]. Previous work has shown that KRAS mutations in NSCLC are positively correlated with PD-L1 expression [14, 15], and patients with only KRAS mutations will have better benefits from immunotherapy [16, 17].

In this research, we evaluated the significance of secretory or membrane-associated protein gene expression and prognosis in relation to KRAS mutation in LUAD. We investigated infiltration characteristics in the TME and their interrelationships with 24 infiltrating immune cells. In addition, a KRAS prediction model was developed utilizing the aforementioned genes to explore the association between KRAS mutation and immune infiltration and to discover promising predictors for KRAS LUAD patients who underwent immunotherapy.

## 2. Materials and Methods

### 2.1. Ethics Statement

The study was approved by the Ethics Committee of Zhongshan Hospital, Fudan University (B2020-035).

### 2.2. Data Preprocessing

We downloaded gene expression data (FPKM format), gene mutations, clinical statistics, and survival data from The Cancer Genome Atlas (TCGA) database (https://gdc.xenahubs.net) [18]. We obtained frozen samples of the patients' normal lung and tumor tissue, omitting recurring tumor tissue, paraffin-embedded cancer samples, and metastatic cancer samples. Patient records lacking survival information were removed (*n* = 22). The Human Protein Atlas (https://www.proteinatlas.org) [19, 20] data on gene localization was retrieved. Gene locations, including their main location, additional location, and extracellular location, are all included in the data that was retrieved from the database. We chose proteins with “plasma membrane” as their main location and additional location with “predicted to be secreted.”

Thirty patients with LUAD who underwent surgery from December 2020 to December 2021 at the Department of Thoracic Surgery, Zhongshan Hospital, Fudan University, were selected. The genetic testing information of the 30 patients was obtained. All participants signed an informed consent form in accordance with the Helsinki Declaration's ethical criteria. Ethics is authorized by Zhongshan Hospital's ethical committees (B2020-035).

### 2.3. Differentially Expressed Secretory or Membrane-Associated Gene Analysis

Limma packages [20] in the R software were used to analyze the differentially expressed genes (DEGs) among the KRAS mutant, the wild-type (WT), and the normal groups (Version 3.7.2). We further explored secretory or membrane-associated DEGs in the KRAS mutation subgroup (G12C, G12V, and other mutation subtypes). We established the criteria as the adjusted *p* value < 0.05 and log2 fold change [21] >0.3. The R packages ggplot2 and pheatmap depict the result of differential gene standardized expression values.

### 2.4. Survival Analysis

We showed the overall survival (OS) difference of differentially expressed secretory or membrane-associated protein genes using a Kaplan-Meier survival curve constructed by the ggplot2 package and set the *p* value < 0.05 to indicate a statistically significant difference. The log-rank test and COX analysis were used to demonstrate the differences.

### 2.5. Functional Enrichment Analysis

The R package “cluster profile” was used to conduct gene ontology (GO) and Kyoto Encyclopedia of Genes and Genomes (KEGG) enrichment analysis on differentially expressed secretory or membrane-associated genes, respectively, to demonstrate the relevance of prognosis. Adjusted *p* value < 0.05 and false discovery rate (FDR) < 0.05 define the threshold for GO and KEGG analysis.

### 2.6. Identification of the Infiltrating Immune Cells

Based on earlier studies [22, 23], we used the gene markers described by Bindea et al. [24]. A gene profile related to the immunological microenvironment cell sets encompassing 585 genes was created, and 24 tumor microenvironment (TME) infiltrating cell types associated with innate immunity and adaptive immunity were shown: B cells, dendritic cells (DCs), immature dendritic cells, activated dendritic cells, neutrophils, macrophages, natural killer (NK) cells, natural killer-CD56 cells, mast cells, natural killer-CD56dim cells, cytotoxic cells, eosinophils, T cells, CD8+ T cells, Th1, Th2, Th17, follicular helper T cell (TFH), Tgd, T, T-helper, and central memory T cells (Tcm). Gene Set Variation Analysis (GSVA) [25, 26] was used to evaluate the extent of immune cell infiltration and has the advantage of standardizing gene expression and minimizing data noise [27]. For the purpose of determining the characteristics between differentially expressed secretory or membrane-associated genes and infiltrating immune cells, the infiltration enrichment scores of each immune cell type in normal lung and LUAD samples were adjusted (from 0 to 1). The pheatmap packages displayed the correlations via heatmaps.

### 2.7. Construction of the KRAS Mutation Predictive Model

We conducted least absolute shrinkage and selection operator- (LASSO-) logistic [28] regression analysis based on the secretory or membrane-associated protein DEGs for tenfold cross-validation using the glmnet [29] package in R to develop a model for predicting KRAS mutation in patients with LUAD. *λ* is the tuning parameter, which was established based on the minimal number of genes and the 1-SE criterion.

Using R's rms package, a nomogram [30] of 505 lung adenocarcinoma samples from TCGA was constructed. The survival-related parameters of LUAD patients were displayed using a visual regression model, which included clinical-pathological features and eight selected secretory or membrane-associated genes from LASSO analysis. For the preceding model, the concordance index (C-index) and the calibration graph curve show the accuracy of the prediction.

### 2.8. Extraction of RNA and Real-Time Quantitative Polymerase Chain Reaction

As an RNA extraction reagent, TRIzol (Tiangen Biotechnology Co, Beijing, China) was used. The cDNA template was synthesized using a PrimeScript RT Reagent Kit (TaKaRa, Tokyo, Japan), and quantitative real-time polymerase chain reaction was conducted using SYBR Premix Ex Taq (TaKaRa) according to the manufacturer's instructions. Each reaction was assessed using QuantStudio 5 (Thermo Fisher Scientific). To quantitatively quantify the mRNA, the 2-CT method with GAPDH as the endogenous calibrator was used. The sequences of all primers were produced by Sangon Biotech.

## 3. Results

### 3.1. Selection for Secretory or Membrane-Associated DEGs

**Figure 1:**
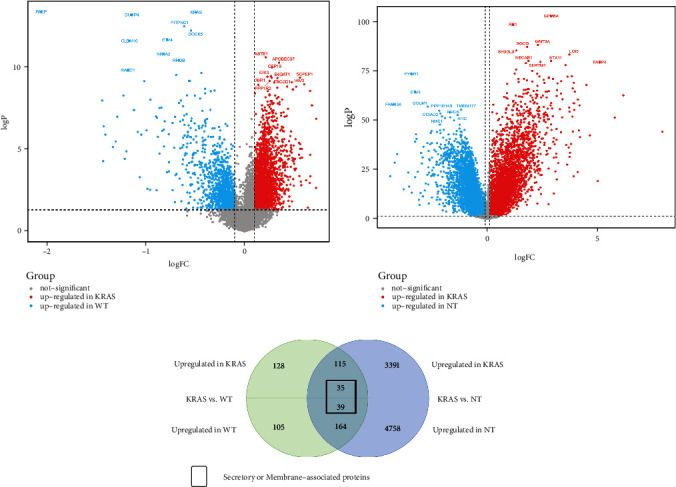
Selection for secretory or membrane-associated DEGs: (a) the volcano plot showed the differentially expressed genes in KRAS vs. WT group; (b) the volcano plot showed the differentially expressed genes in KRAS vs. NT group; (c) the Wayne diagram showed selection for secretory or membrane-associated DEGs.

The 563 LUAD samples were divided into three groups, the KRAS mutated group (*n* = 137), the wild-type (WT) group (*n* = 368), and the nontumor group (NT) (*n* = 58). There are 54 G12C, 30 G12V, and 53 other mutation types in the KRAS mutated group. Differentially expressed genes were analyzed between the KRAS mutation group vs. WT group and the KRAS mutation group vs. NT group. In the first comparison, 278 genes were upregulated in the KRAS mutation group, while 308 genes were upregulated in the WT group (Figure 1(a)). In the KRAS vs. NT group, 3541 genes were upregulated in the KRAS group, and 4961 genes were upregulated in the NT group (Figure 1(b)). We matched the DEGs upregulated in the KRAS mutated group to get 150 genes, while the DEGs upregulated in the WT group were matched with those upregulated in the NT group, and 203 genes remained. Seventy-four of the proteins expressed by these 353 genes are secretory or membrane-associated proteins, including HMGCS1, RNF39, IL37, INSR, and KIF12 (all adjusted *p* < 0.05 and |log2 fold change| [21] >0.3) (Figure 1(c)).

**Figure 2:**
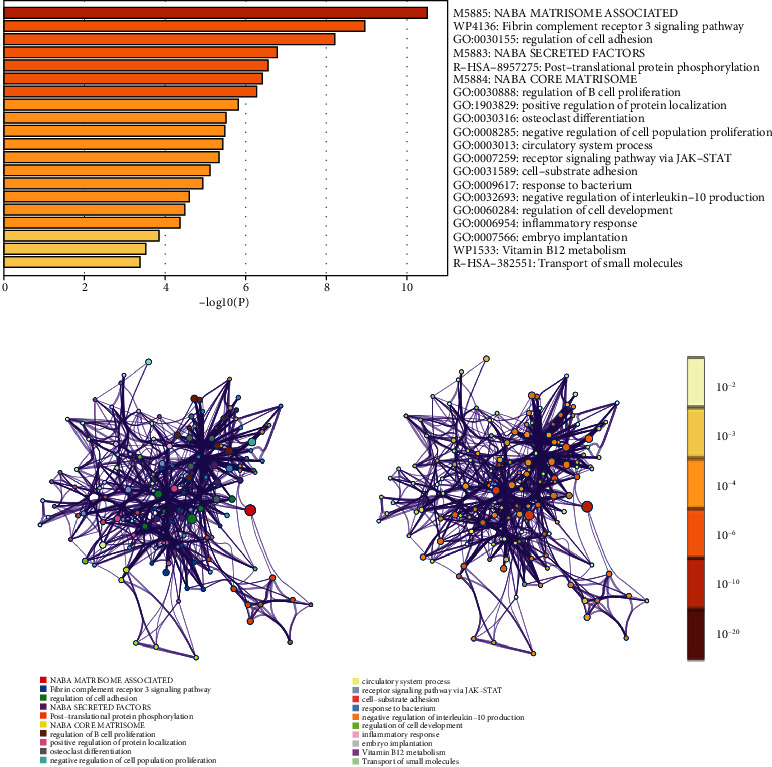
(a) GO and KEGG functional enrichment analyses of the secretory or membrane-associated DEGs; (b) interactions between the enriched pathways among 74 genes. The size of the dots indicates the number of genes in the relevant pathway, and the color indicates the cluster category. The pathways for the clustering category are displayed in the tab on the label. Dots representing the same enrichment pathway have different colors depending on the *p* value displayed on labels. The darker the color, the smaller the *p* value, and the more statistically significant it is.

GO and KEGG functional enrichment analysis of these secretory or membrane-associated DEGs (*n* = 74) was conducted to explore their function (Figure 2). The results show that the most relevant pathways, including cell activation, osteoclast differentiation, and regulation of cellular location, were highly correlated with immune cell infiltration and tumor immune microenvironment. The secretory or membrane-associated DEGs were also enriched in pathways like the transmembrane receptor protein tyrosine kinase signaling pathway, which was also correlated with TME.

### 3.2. Secretory or Membrane-Associated DEGs Associated with Survival in KRAS Mutated Group and KRAS Subgroups

**Figure 3:**
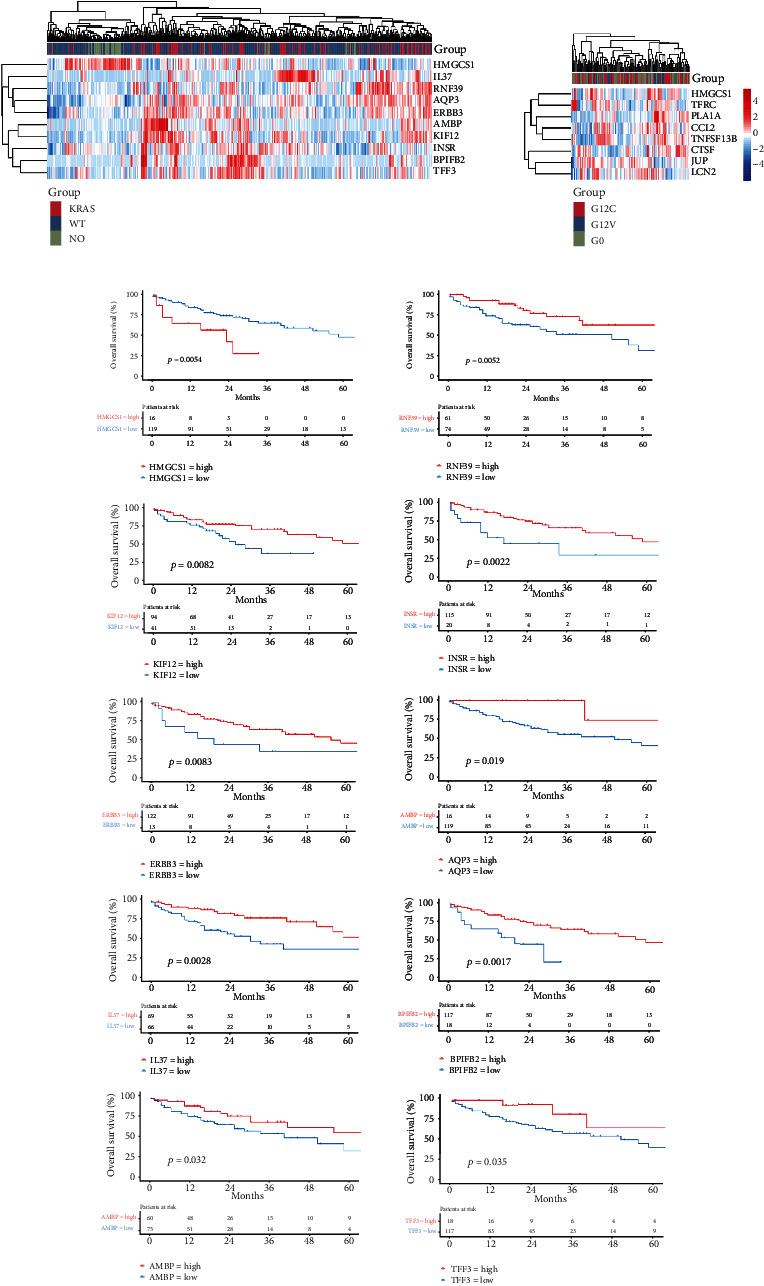
(a) Left: the heatmaps of the survival-related secretory or membrane-associated protein DEGs. Right: the heatmaps of the secretory or membrane-associated protein DEGs among KRAS mutation subgroups. (b) Kaplan-Meier curves of OS about ten genes in LUAD patients with KRAS mutation.

To further investigate possible targets for predicting immunotherapy efficiency in patients with KRAS-mutated LUAD, we studied those above 74 secretory or membrane-associated DEGs that were substantially related to prognosis in KRAS LUAD patients and DEGs in subgroups in the KRAS mutation group. The gene sets contained ten genes: HMGCS1 was related to poorer survival (HR > 1) and upregulated in the KRAS group. The other nine genes, RNF39, KIF12, INSR, ERBB3, AQP3, IL37, BPIFB2, AMBP, and TFF3, functioned as tumor suppressor genes (HR < 1) and were downregulated in the KRAS group. Further DEG analysis was performed in the KRAS mutation subgroups (the G12C, G12V, and other mutation groups were compared in two pairs) to obtain a gene set containing eight genes. HMGCS1 was associated with a worse prognosis. Heatmaps depicted the expression characteristics of the ten genes in the LUAD sample (Figure 3(a), left), as well as the eight genes among the KRAS subgroups (Figure 3(a), right). The survival analysis of the ten genes revealed a significant difference in the KRAS mutated group (*n* = 137) (Figure 3(b)).

### 3.3. Experimental Validation of Secretory or Membrane-Associated DEGs Associated with KRAS Mutated Group

**Figure 4:**
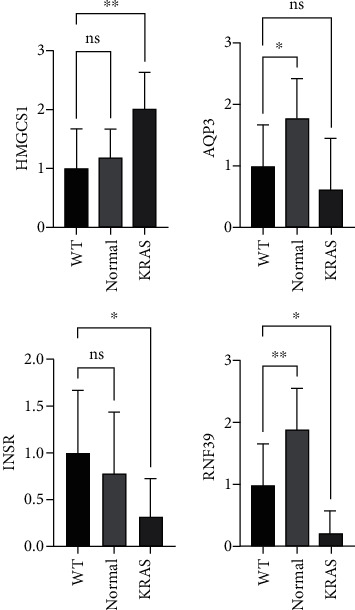
Quantitative RT-PCR verifying the expression of HMGCS1 INSR, RNF39, and AQP3 in the KRAS group, the WT group, and normal paracancerous tissues. ^∗^*p* < 0.05; ^∗∗^*p* < 0.01; ^∗∗∗^*p* < 0.001; ^∗∗∗∗^*p* < 0.0001.

We obtained the genetic testing results of the 30 patients with LUAD from our hospital. Among them, ten patients have KRAS mutations and 20 cases have wild type. We extracted tissue RNA from the KRAS mutated group and the WT group, as well as their normal paracancerous tissues. The results of qPCR showed that the gene HMGCS1 was overexpressed in the KRAS group compared to normal tissue, while genes INSR, RNF39, and AQP3 had decreased expression in the KRAS group (Figure 4), which was also consistent with the results of differential gene analysis.

### 3.4. Characterization between Infiltrating Immune Cells and the Secretory or Membrane-Associated DEGs

We investigated the characterization between prognosis-associated secretory or membrane-associated protein DEGs and the 24 immune cell subsets in the tumor immune microenvironment using LUAD data. We calculated the scores of 24 immune cells via GSVA.

**Figure 5:**
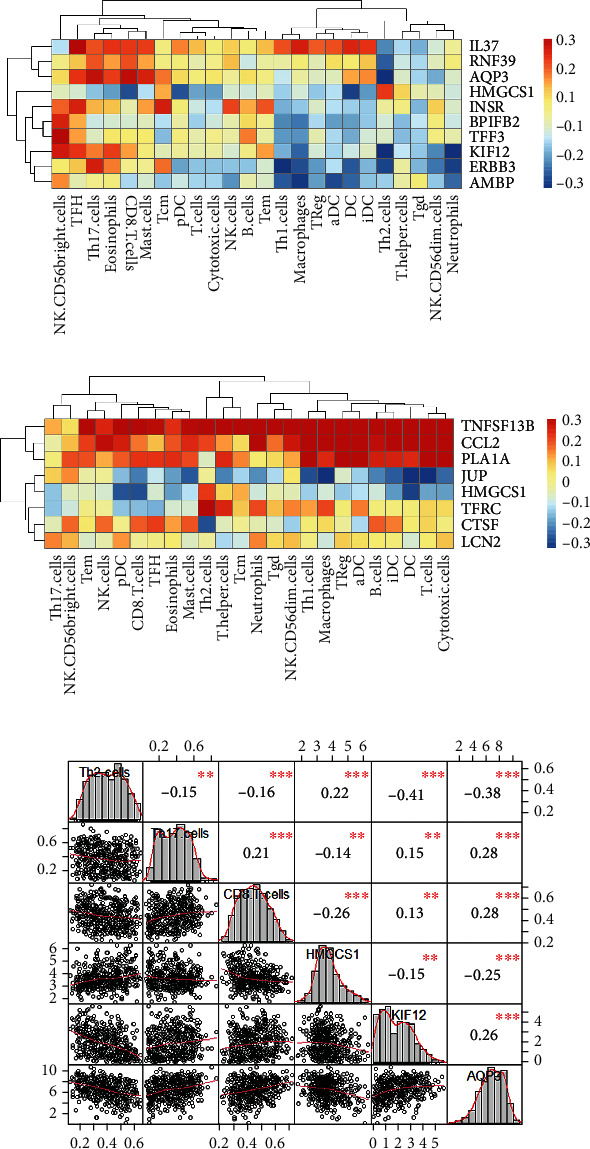
Correlations between secretory or membrane-associated DEGs and immune cells in the tumor microenvironment: (a) correlations between survival-associated DEGs and infiltrating immune cells; (b) correlations between DEGs from KRAS subgroups and infiltrating immune cells; (c) correlations among Th2, Th17, CD8+ T cell, HMGCS1, KIF12, and AQP3.

IL37 is highly correlated with most immune cells and most associated with TFH. KIF2 was substantially related to NK.CD56bright among the ten genes. INSR was associated with TFH and Tcm cells, while AQP3 was related to CD8+ T cells and Th17 cells. Other secretory or membrane-associated DEGs have little associations with immune cells (Figure 5(a)). To further study the relationship between immune cells and SMP genes, we analyzed the correlation and showed a significant positive correlation between AQP3 and Th17 and CD8+ T cells (Figure 5(c)). In DEG sets from the KRAS-mutant subgroup, TNFSF13B and CCL2 were highly related to infiltrating immune cells (Figure 5(b)). Both activated immune cells like NK, TFH, cytotoxic cells, T cells, Th1.cells, and suppressed cells like regulatory T cells (Treg) were highly related to secretory or membrane-associated DEGs.

Interestingly, NK.CD56bright cells showed a high correlation with gene sets from KRAS survival-related group while having a poor relationship with gene sets from KRAS mutation subgroups. Also, HMGCS1 is the intersection of the two gene sets, but it has little correlation with infiltrating immune cells. The above characteristics indicated that the expression of the genes played distinct roles in the infiltration of immune cells into the immune environment.

### 3.5. Construction of the KRAS Mutation Prediction Model

**Figure 6:**
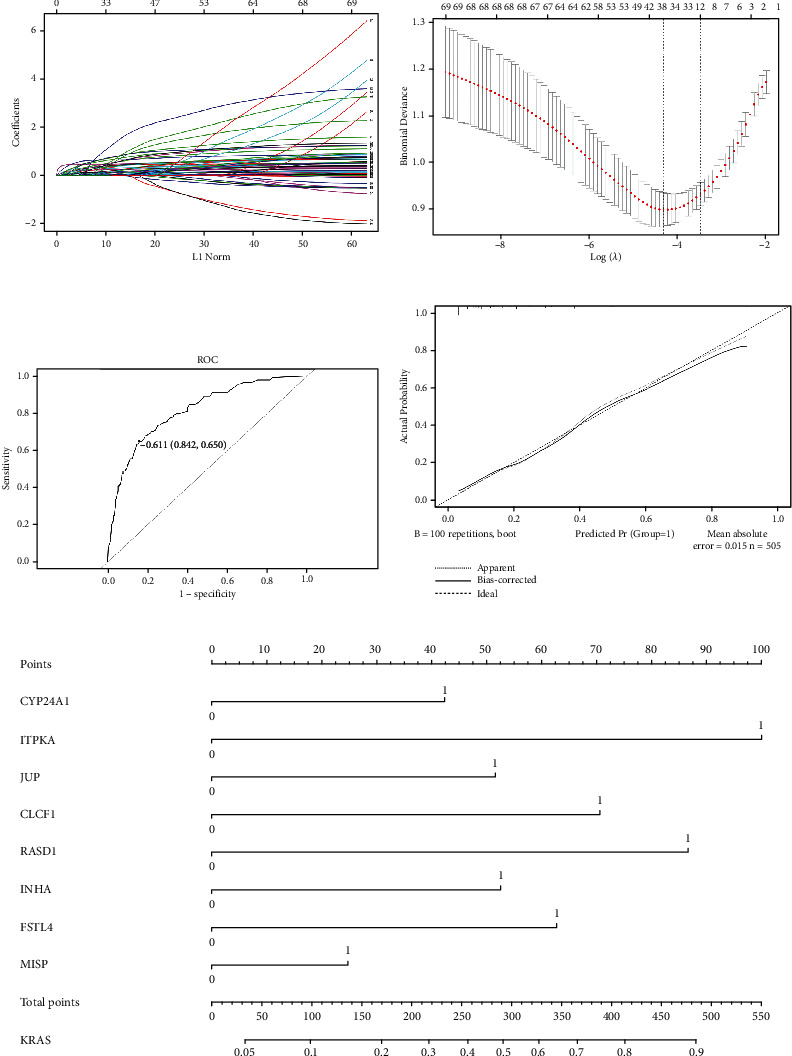
(a) Coefficient profiles of variables in the LASSO regression model; (b) tenfold cross-validation for selecting parameter *λ* in the LASSO regression model; *λ* is the turning parameter. The partial likelihood deviance is plotted in log(*λ*), in which vertical lines are shown at the optimal values by minimum criteria and 1−SE criteria; (c) ROC curve of the prediction model; (d) calibration plot for nomogram; (e) nomogram for the prediction model.

**Figure 7:**
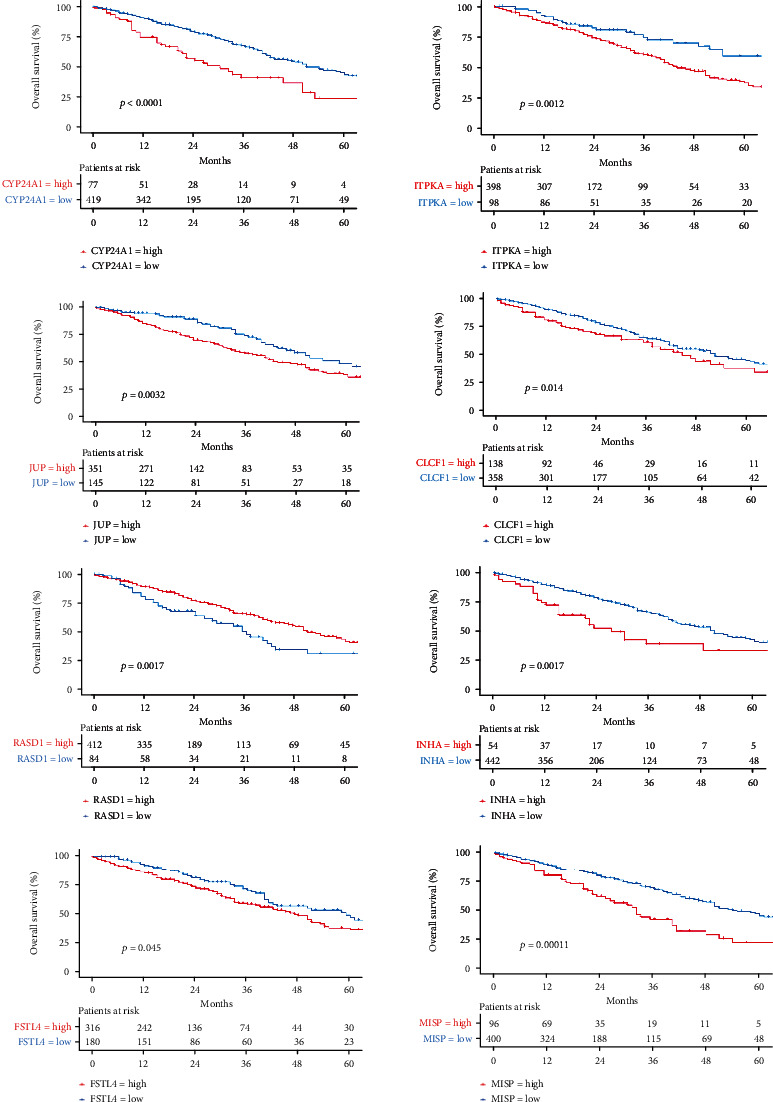
Kaplan-Meier curves of OS about eight genes in LUAD patients from TCGA.

Eight genes are selected to construct the KRAS mutation prediction model, including CYP24A1, ITPKA, JUP, CLCF1, RASD1, INHA, FSTL4, and MISP. LASSO-logistic regression analysis can reduce model distortion caused by the effects of multiple collinearities (Figures 6(a) and 6(b)). All eight genes were substantially associated with survival in patients with LUAD (*p* < 0.05). COX regression analysis revealed that all eight genes were related to poorer survival (Figure 7). On the basis of the logistic regression coefficient and the eight genes listed above, we built a KRAS mutation prediction model: score = (0.4825) × CYP24A1 + (1.1393) × ITPKA + (0.5873) × JUP + (0.8046) × CLCF1 + (0.9878) × RASD1 + (0.5984) × INHA + (0.7144) × FSTL4 + (0.2822) × MISP − 3.3221.

Under this predictive model, each LUAD patient from TCGA (*n* = 505) would receive a score based on the expression of eight genes, with the threshold being determined using the ROC curve approach. Using the cutoff value, the patient's score was separated into two categories: mutation and wild type (Figure 6(c)). The predictive accuracy of the model is 0.79. Based on the results of the logistic regression analysis, we plotted (Figure 6(e)) a nomogram depicting a model of eight genes capable of predicting KRAS mutation in LUAD patients. The calibration curve revealed a high degree of coincidence and high prediction accuracy (Figure 6(d)).

## 4. Discussion

In this study, we downloaded cell-specific localization data of LUAD and normal tissues from The Human Protein Atlas and explored the expression of KRAS-related secretory or membrane-associated proteins in LUAD samples. According to the results of the GO and KEGG analyses, these genes have close correlations to the infiltrating immune cells and immunological microenvironment pathways. We discovered ten differentially expressed secretory or membrane-associated protein genes that were substantially correlated with the prognosis of KRAS-mutant LUAD patients and eight DEGs among subgroups of KRAS mutation. We confirmed the characterization of their interactions with immune cells and roles in immune infiltration. We hope to identify a potential immunotherapy prediction target for patients with KRAS-mutated lung cancer.

We explored the characteristics of correlations between secretory or membrane-associated proteins and infiltrating immune cells in KRAS LUAD patients. TME is composed of immune cells, fibroblasts, blood vessels, and lymphoid tissue surrounding tumor cells and has various abilities to stimulate tumorigenesis to inhibit tumor formation [31]. Immunotherapy effectiveness is correlated with the level of infiltration of vital immune cells such as CD8+ T lymphocytes, B cells, dendritic cells, and macrophages. PD-1 is a membrane-associated protein expressed in T cells [10]. Both CD8+ T lymphocytes and Treg cells contain PD-1, which interacts with T cell membrane receptors and regulates the immune system [32, 33]. The two PD-L1 ligands are crucial dendritic cell costimulating ligands [34, 35]. The binding of dendritic cell ligands to T cell membrane receptors is vital in initiating T cell activity. Exploring the function of secretory or membrane-associated proteins and immune cells is crucial for predicting the efficiency of immunotherapy and revealing its mechanism.

Our study identified ten secretory or membrane-associated genes differentially upregulated in the KRAS mutant group compared to the WT or NT group and were closely related to the prognosis of KRAS LUAD patients. As further analysis showed, eight secretory or membrane-associated DEGs among subgroups of KRAS mutation were identified, which were highly related to infiltrating immune cells. TNSF13B, IL37, KIF2, INSR, and AQP3 were closely correlated with infiltrating immune cells. Human B cell activator factor (TNFSF13B, BAFF) is a member of the tumor necrosis factor superfamily, which mainly mediates gene expression in B cells, regulates T cells [36], has previously reported high expression in the immune microenvironment of multiple tumors [37], and is highly correlated with Treg and CD8+ T cells, which showed the highest correlation with infiltrating immune cells in our study. Interleukin 37 (IL37) is an immunosuppressive factor demonstrated in previous studies to inhibit cell proliferation by inhibiting the STAT3 pathway of TFH cells [13]. The kinesin family (KIF), which played a crucial role in microtubule function [38] and has been associated with the growth of multiple tumors mediating cell mitosis and gene mutation [39], was found to be closely associated with the formation of various tumors. KIF2A induces tumor cell proliferation by regulating the PI3K/Akt pathway [40]. By suppressing membrane-type 1-matrix metalloproteinase, KIF2A knockdown in gastrointestinal cancers decreases tumor cell invasion [41]. Although various lncRNAs, such as the LINC00958/miR-204-3p/KIF2A axis [42], play a significant role in the development of NSCLC, its interaction with immune cells in NSCLC warrants additional investigation. The aquaporin 3 (AQP3) gene in NSCLC cells mediates the adhesion of floating cells by the action of protrusions on the cell surface, hence increasing the aggregation of tumor cell invasion [43]. Zhu et al. [44] confirmed that APQ3 plays a vital function in macrophage immunity by stimulating phagocytosis and migration. Moreover, it mediates the migration of T cells via cytokines in the T cell immune response [45], which may explain its close link with immune cell infiltration and its function in forming LUAD.

In the KRAS mutant prediction model, eight genes closely associated with the prognosis of LUAD patients were analyzed to develop a predictive model. Three of them play an important role in the occurrence and development of LUAD. Previous research shows that overexpression of cytochrome P450 family 24 subfamilies A member 1 (CYP24A1) enhances lung cancer cell proliferation through activating RAS signaling [46]. ITPKA (inositol 1,4,5-trisphosphate 3-kinase), a member of the inositol polyphosphate kinase (IPK) family, promotes LUAD by interacting with Drebrin 1 and triggering the epithelial-mesenchymal transition (EMT) [47]. Kim et al. [48] found the functional significance of the cardiotrophin-like cytokine factor 1-ciliary neurotrophic factor receptor (CLCF1-CNTFR) signalling axis in lung adenocarcinoma (LUAD) and engineered a high-affinity soluble receptor (eCNTFR-Fc) that sequesters CLCF1, thereby inhibiting its oncogenic effects. They detected a link between the efficacy of eCNTFR-Fc and the existence of KRAS mutations that maintain the inherent potential to hydrolyze guanosine triphosphate, indicating that the mechanism of action may be associated with altered guanosine triphosphate loading.

This study still has several limitations. First, we only analyzed LUAD patient samples from TCGA. Validation should be performed in larger sample sizes in the GEO database. Besides, LUAD patients with KRAS mutations alone and STK11 comutations responded differently to the efficacy of immunotherapy [49]. Also, patterns of immune cell infiltration of the KRAS-mutated group and wild-type group were not analyzed. We validated the gene expression changes of HMGCS1 INSR, RNF39, and AQP3 in the KRAS mutant, WT, and normal groups by PCR, and future studies should focus on verifying gene changes in patients' immune infiltrating cells. Furthermore, because LASSO-logistic regression is for bivariate analysis, the prediction model is more suitable for predicting KRAS mutation or not, and since there are many types of KRAS mutation subgroups, but the sample number is relatively small, a more appropriate model for the prediction of KRAS mutation subgroups needs to be selected, which is worth further investigation. Similarly, for predicting TME mutation types, the predictive model is less applicable to trichotomy and requires additional screening for genes highly associated with immunity [50]. The English usage of the article needs to be further polished.

## Data Availability

Data can be obtained from the UCSC Xena browser (https://gdc.xenahubs.net).
